# Medical Nutrition Therapy in Dermatological Diseases: A Joint Consensus Statement of the Italian Association of Dietetics and Clinical Nutrition (ADI), the Italian Society of Dermatology and Sexually Transmitted Diseases (SIDeMaST), the Italian Society of Nutraceuticals (SINut), *Club Ketodiets and Nutraceuticals “KetoNut-SINut”* and the Italian Society of Endocrinology (SIE), *Club Nutrition, Hormones and Metabolism*

**DOI:** 10.1007/s13679-025-00630-2

**Published:** 2025-05-13

**Authors:** Luigi Barrea, Ludovica Verde, Giuseppe Annunziata, Emiliano Antiga, Elisabetta Camajani, Massimiliano Caprio, Maria Grazia Carbonelli, Augusto Carducci, Edda Cava, Giorgia Di Marco, Davide Grassi, Stefania Guida, Barbara Martinelli, Angelo Valerio Marzano, Chiara Moltrasio, Massimiliano Petrelli, Francesca Prignano, Franco Rongioletti, Silvia Savastano, Barbara Paolini, Carmela Bagnato, Giuseppe Argenziano, Arrigo Francesco Giuseppe Cicero, Annamaria Colao, Diego Ferone, Gianluca Aimaretti, Giovanna Muscogiuri

**Affiliations:** 1 Dipartimento Di Psicologia E Scienze Della Salute, Università Telematica Pegaso, Centro Direzionale, Via Porzio, Isola F2, 80143 Naples, Italy; 2 Department of Public Health, University of Naples Federico II, Via Sergio Pansini 5, Naples, Italy; 3 Department of Medicine, Division of Endocrinology, University of Arizona, Tucson, AZ USA; 4 Facoltà Di Scienze Umane, Della Formazione E Dello Sport, Università Telematica Pegaso, Via Porzio, Centro Direzionale, Isola F2, 80143 Naples, Italy; 5 Department of Health Sciences, Section of Dermatology, University of Florence, Florence, Italy; 6 Laboratory of Cardiovascular Endocrinology, IRCCS San Raffaele, Rome, Italy; 7 Department for the Promotion of Human Sciences and Quality of Life, San Raffaele Roma Open University, Via Di Val Cannuta 247, 00166 Rome, Italy; 8 Clinical Nutrition and Dietetics, San Camillo Forlanini Hospital, Rome Cir.Ne Gianicolense 87, 00152 Rome, Italy; 9 Internal Medicine Unit—Val Vibrata Hospital—Sant’Omero (TE)—Department of Life, Health and Environmental Sciences, University of L’Aquila, L’Aquila, Italy; 10 Department of Dermatology, Vita-Salute San Raffaele University, Milan, Italy; 11 Department of Medical Sciences, Unit of Dietetics and Clinical Nutrition, Santa Maria Alle Scotte Hospital, University of Siena, 53100 Siena, Italy; 12 Dermatology Unit, Fondazione IRCCS Ca’ Granda Ospedale Maggiore Policlinico, 20122 Milan, Italy; 13 Department of Pathophysiology and Transplantation, Università Degli Studi Di Milano, 20122 Milan, Italy; 14 Clinic of Endocrinology and Metabolic Diseases, Polytechnic University of Ancona, 60100 Ancona, Italy; 15 Department of Health Sciences, Section of Dermatology, University of Florence, Ancona, Italy; 16 Dipartimento Di Medicina Clinica E Chirurgia, Unità Di Endocrinologia, Università Degli Studi Di Napoli Federico II, Naples, Italy; 17 Centro Italiano Per La Cura E Il Benessere del Paziente Con Obesità (C.I.B.O), Dipartimento Di Endocrinologia, Diabetologia, Andrologia e Nutrizione, AOU Federico II, Via Sergio Pansini 5, 80131 Naples, Italy; 18 UOSA of Dietetics and Clinical Nutrition, Azienda Ospedaliera Universitaria Senese, Policlinico Santa Maria Alle Scotte, Siena, Italy; 19 UOSD Clinical Nutrition and Dietetic, Hospital Matera, 75100 Matera, Italy; 20 Dermatology Unit, University of Campania “L. Vanvitelli”, Naples, Italy; 21 Hypertension and Cardiovascular Risk Factors Research Unit, Medical and Surgical Sciences Dept, Alma Mater Studiorum University of Bologna, Via Massarenti 9, 40138 Bologna, Italy; 22 Cardiovascular Medicine Unit, IRCCS AOU Di Bologna, Bologna, Italy; 23 Cattedra Unesco “Educazione Alla Salute E Allo Sviluppo Sostenibile”, University Federico II, Naples, Italy; 24 Endocrinology Unit, Department of Internal Medicine and Medical Specialties, School of Medical and Pharmaceutical Sciences, University of Genova, 16132 Genoa, Italy; 25 Endocrinology, Department of Translational Medicine, Università del Piemonte Orientale, Novara, Italy

**Keywords:** Acne, Hidradenitis suppurativa, Psoriasis, Medical nutrition therapy, Diet, Nutrition, Obesity, Mediterranean diet, Ketogenic diet, VLEKT

## Abstract

**Summary:**

Dermatological diseases such as acne, hidradenitis suppurativa (HS), and psoriasis are driven by chronic inflammation and oxidative stress. Emerging evidence highlights the role of nutrition in modulating these conditions, particularly through dietary patterns rich in antioxidants, polyphenols, and unsaturated fatty acids.

**Recent Findings:**

The Mediterranean diet (MedDiet) has demonstrated potential benefits due to its anti-inflammatory and immunomodulatory effects, while very low-energy ketogenic therapy (VLEKT) has shown promise in rapidly improving disease severity. Specific nutrients, including omega-3 fatty acids, probiotics, and micronutrients, may further contribute to disease management. However, the current literature is limited by small-scale studies and the lack of standardized dietary guidelines.

**Purpose of Review:**

This *Consensus Statement*, developed collaboratively by the Italian Association of Dietetics and Clinical Nutrition (ADI), the Italian Society of Dermatology and Sexually Transmitted Diseases (SIDeMaST), the Italian Society of Nutraceuticals (SINut), *Club Ketodiets and Nutraceuticals “KetoNut-SINut”* and the Italian Society of Endocrinology (SIE), *Club Nutrition, Hormones and Metabolism*, aimed to establish an evidence-based framework for medical nutrition therapy (MNT) of the most common inflammatory skin diseases, including acne, HS and psoriasis.

## Introduction

Dermatological diseases, such as acne, hidradenitis suppurativa (HS) and psoriasis, are often characterized by chronic inflammation and oxidative stress, both of which play central roles in their pathophysiology [1–3]. In acne, the overproduction of sebum, follicular hyperkeratinization, and bacterial colonization are exacerbated by pro-inflammatory mediators and oxidative damage, contributing to lesion formation [4]. Similarly, psoriasis, a multifactorial inflammatory disease, is marked by the dysregulation of immune pathways, particularly the overactivation of helper T cell (Th) 17 and Th1 cells, which perpetuate oxidative damage to keratinocytes [5]. HS, a chronic relapsing skin condition, shares these inflammatory and oxidative hallmarks, with additional contributions from dysregulated adipokines and microbial imbalance [6].

Obesity and its associated comorbidities further amplify this inflammatory and oxidative burden, creating a vicious cycle that worsens dermatological diseases [7]. Adipose tissue, especially in central obesity, serves as a potent source of pro-inflammatory cytokines, such as tumor necrosis factor-alpha (TNF-α) and interleukin (IL)− 6. These cytokines not only contribute to systemic inflammation but also directly impact skin homeostasis, exacerbating conditions like acne, HS and psoriasis [7]. Furthermore, metabolic comorbidities, including insulin resistance and dyslipidemia, increase oxidative stress [8], compromising skin barrier function and immune regulation [1].

The complex interplay among nutrition, inflammation, and oxidative stress has become a major focus of research in recent years across different clinical settings and patient populations [9]. Diets high in refined carbohydrates, saturated fats, and dairy products have been implicated in the exacerbation of inflammatory dermatological conditions, primarily through mechanisms involving hyperinsulinemia and heightened insulin-like growth factor- 1 (IGF- 1) activity [10]. Conversely, plant-based dietary patterns, such as the Mediterranean diet (MedDiet), are increasingly recognized for their protective role due to their richness in antioxidants, polyphenols, and unsaturated fatty acids [10]. Recent evidence also highlights the potential of the very low-calorie ketogenic diet (VLCKD), recently renamed very low-energy ketogenic therapy (VLEKT) [11], not only for obesity and metabolic diseases [12] but also for dermatological diseases such as acne [13], HS [14], and psoriasis [15]. Additionally, some nutraceuticals, including omega- 3 fatty acids, probiotics, and specific micronutrients, have shown potential in modulating the severity of dermatological diseases, likely through the regulation of key inflammatory and oxidative pathways [16, 17]. However, despite these advancements, the lack of standardized guidelines on dietary strategies for dermatological diseases remains a critical gap.

Recognizing this need, this *consensus statement*, developed collaboratively by the Italian Association of Dietetics and Clinical Nutrition (ADI), the Italian Society of Dermatology and Sexually Transmitted Diseases (SIDeMaST), the Italian Society of Nutraceuticals (SINut), *Club Ketodiets and Nutraceuticals “KetoNut-SINut”* and the Italian Society of Endocrinology (SIE), *Club Nutrition, Hormones and Metabolism*, aimed to establish an evidence-based framework for medical nutrition therapy (MNT) of the common inflammatory skin diseases, including acne, HS and psoriasis.

## Medical Nutrition Therapy in Acne

### Pathophysiology of Acne

Acne vulgaris is a multifactorial disorder, involving both genetic and environmental factors, affecting the pilosebaceous unit [4]. It has been related to the interplay among follicular hyperkeratinization, sebaceous gland dysfunction, hyperproliferation of *Cutibacterium acnes*, and inflammation, further exacerbated by oxidative stress, and modulated by several factors including hormones and metabolic alterations [4]. Hormonal imbalances associated with acne lead to increased androgen levels, which, in turn, increase sebum production and promote keratinocyte proliferation [18]. This proliferation, together with abnormal differentiation of follicular epithelial cells observed in patients with acne, leads to follicular hyperkeratinization [18].

Additionally, lipid oxidation, particularly of squalene, generates lipoperoxides that trigger inflammatory cascade responses [19]. Sebum oxidation also creates a microaerophilic environment supporting *Cutibacterium acnes* growth, contributing to microbiota dysregulation. In turn, bacteria activate toll-like receptors on sebocytes and keratinocytes, leading to pro-inflammatory IL- 6, IL- 8, and IL- 12 release and produce porphyrins, inducing reactive oxygen species (ROS) production [20]. This leads to neutrophil recruitment and aggregation, resulting in additional release of ROS and perpetuating inflammation in a self-reinforcing cycle. Lipid peroxidation products also contribute to further disruption of follicular homeostasis [19].

Metabolic factors, including insulin resistance and hyperinsulinemia, further exacerbate acne by stimulating sebocyte hyperactivity and androgen synthesis, linking acne to obesity and metabolic syndrome (MetS) [21–23]. Dyslipidemia also amplifies oxidative stress and inflammation, reinforcing the acne-obesity-MetS axis [21–23].

#### Take Home Message

Acne vulgaris is driven by follicular hyperkeratinization and sebaceous gland dysfunction, with lipid oxidation and skin microbiota dysregulation contributing to impaired follicular homeostasis, inflammation and oxidative stress. Metabolic alterations, such as obesity, further exacerbate these pathogenetic processes through hormonal imbalances, insulin resistance, and dyslipidemia, amplifying inflammation and oxidative stress.

### The Role of Hormones in Acne

It is well established that endocrine mechanisms play a pivotal role in the etiopathogenesis of acne [24]. Several different hormones and growth factors, including androgens, estrogens, glucocorticoids, insulin, IGF- 1, fibroblast growth factors, and epidermal growth factors, are involved in the pathogenesis and the progression of acne, through their binding with high-affinity receptors [24]. Androgens, mainly testosterone and its active metabolite 5α-dihydrotestosterone (5α-DHT) that is converted via 5α-reductase type 1, an enzyme expressed mainly in facial sebocytes and sweat glands, represent the most significant endogenous contributors to the development and maintenance of acne [25]. Indeed, androgen levels correlate with acne severity, and many endocrinopathies characterized by hyperandrogenism, such as polycystic ovary syndrome, late congenital adrenal hyperplasia, and ovarian, adrenal, or pituitary tumours, are associated with acne [25]. However, current experimental research on the pathophysiology of acne is depicting a more complicated background. In particular, the environmental exposures to endocrine disrupting chemicals have proved to alter the androgen receptor-mediated signal transduction within the pilosebaceous unit [26]. In addition, IGF- 1, acting via the AKT/mTORC1/SREBP1 signalling pathway, not only primarily results in an overproduction of sebum, abnormal keratinization of follicles, and perifollicular inflammation, but it increases also the conversion of testosterone to 5α-DHT. Moreover, sebocytes per se can also synthesize androgens in situ, and the interaction of local active androgen production with circulating IGF- 1 exerts worsens the effects on the pilosebaceous unit [26].

#### Take Home Message

Endocrine mechanisms play a crucial role in the pathophysiology of inflammatory dermatological conditions, with androgens, IGF- 1, and environmental endocrine disruptors contributing to sebaceous gland activity, inflammation, and disease progression. The interplay between systemic hormones and local androgen synthesis further exacerbates these effects, highlighting the complexity of hormonal regulation in skin health.

### Acne and Obesity

The link between acne and obesity is complex and multifaceted, as highlighted by various studies exploring their association [27]. Acne vulgaris, the most common skin disorder in the Western world, affects teens and young adults [28]. One potential connection between obesity, MetS and acne is represented by the role of adipokine-driven inflammatory cytokines, which contribute to systemic inflammation and may exacerbate acne [29]. The rising prevalence of obesity among adolescents raised interest in understanding the interplay between these conditions, yet findings from different studies remain inconsistent [30]. For instance, a population-based retrospective cohort study on 643 preadolescents and age- and sex-matched controls found a positive correlation between higher body mass index (BMI) and acne risk [31]. Preadolescents with acne had a higher median BMI percentile compared to controls (75.0 *vs*. 65.0, respectively), with 16.7% of patients with acne suffering from obesity. Additionally, increasing BMI was associated with a higher likelihood of receiving systemic acne treatment, suggesting a relationship between obesity, acne severity, and therapeutic needs [31]. Conversely, a nationwide cross-sectional study in Israel, which included 600.404 adolescents, found an inverse association between BMI and acne [23]. The prevalence of acne decreased progressively from underweight to obesity. Participants with a severe obesity exhibited the lowest odds of acne (adjusted odds ratio [aOR]: 0.53 for males and 0.50 for females), indicating a potential protective effect of higher BMI in this cohort [23].

Further evidence points to a link between obesity, body composition, and acne severity [32]. A prospective case–control study involving 320 individuals revealed that patients with acne vulgaris had significantly higher BMI, body fat percentage, and fat mass compared to healthy controls. Notably, these parameters were particularly elevated in patients with moderate to severe acne. Female patients demonstrated higher body fat percentages, while male patients showed increased fat-free body mass and total body water. These findings suggest that body composition metrics may predict acne severity and could help identify patients at risk of metabolic complications [32].

#### Take Home Message

While obesity and acne are frequently associated, the relationship appears to vary by populations, age, and sex. Some studies suggest that higher BMI increases the risk and severity of acne, while others indicate a protective effect. These discrepancies highlight the need for further research to clarify the underlying mechanisms.

### Acne and Cardiovascular Risk

Acne vulgaris, traditionally viewed as a localized dermatological condition, is now recognized for its potential systemic implications, including an association with increased cardiovascular risk [33, 34]. This chronic inflammatory skin disorder affects approximately 85% of adolescents and a substantial proportion of adults, with emerging evidence linking it to metabolic dysregulation and cardiovascular comorbidities such as hypertension, dyslipidemia, insulin resistance, and MetS [33, 34].

Acne pathogenesis involves the dysregulation of innate and adaptive immune responses, with increased levels of pro-inflammatory cytokines such as TNF-α, IL- 17 [35]. These inflammatory mediators are also key players in the development of atherosclerosis, endothelial dysfunction, and insulin resistance-critical pathways in cardiovascular diseases (CVDs) [36].

The IGF- 1 and the mTORC1 signaling pathways, which are implicated in acne development, also contribute to metabolic and cardiovascular disorders [37]. IGF- 1 promotes sebaceous gland proliferation and sebum production in acne, while simultaneously influencing lipid metabolism and insulin sensitivity. The mTORC1, involved in lipid synthesis and cellular growth, exacerbates both acne and metabolic dysfunctions that predispose individuals to CVDs [33, 37].

Furthermore, it has been suggested that individuals with acne present frequently with dyslipidemia, probably also under androgenic influence, characterized by elevated total cholesterol, triglycerides, and low-density lipoprotein (LDL) cholesterol, along with reduced high-density lipoprotein (HDL) cholesterol levels [33, 34]. These lipid abnormalities are well-established risk factors for atherosclerosis and subsequent cardiovascular events. Studies on lipid profiles in patients with acne revealed that men, in particular, exhibited significant lipid derangements, including elevated triglycerides and LDL-cholesterol levels [33, 34]. This dyslipidemic profile enhances the atherogenic potential, contributing to the early onset of subclinical atherosclerosis [33, 38]. The higher androgenic hormonal effect in acne, coupled with chronic inflammation, creates a synergistic risk for cardiovascular pathology [33, 38].

Insulin resistance is a common feature in both acne and CVDs. Studies have shown that individuals with acne, especially adult males, exhibit higher fasting insulin levels and impaired glucose tolerance compared to controls [33]. Insulin resistance contributes to endothelial dysfunction, hypertension, and dyslipidemia, all of which are pivotal in the pathogenesis of CVDs [39].

Hyperinsulinemia, a consequence of insulin resistance, exacerbates acne by stimulating androgen production, increasing sebaceous gland activity, and promoting keratinocyte proliferation [39]. Simultaneously, it accelerates atherogenesis through pro-inflammatory pathways and adverse lipid profile alterations [33, 37]. The bidirectional relationship between acne and metabolic disturbances underscores the systemic nature of the disease and its cardiovascular implications [33, 37].

Hypertension, a major cardiovascular risk factor, has been associated with acne, particularly in males with persistent post-adolescent acne [34]. The androgen-driven pathogenesis of acne parallels mechanisms contributing to elevated blood pressure [40]. Androgens influence vascular tone by modulating the renin–angiotensin–aldosterone system (RAAS), enhancing sodium retention, and increasing sympathetic nervous system activity [33, 40].

Inflammatory cytokines such as IL- 6 and TNF-α, elevated in acne, also play a role in hypertension [33, 40]. IL- 6 promotes vascular inflammation and stiffness, while TNF-α contributes to endothelial dysfunction and arterial remodeling. These overlapping pathways highlight the inflammatory-hormonal axis linking acne to hypertension and broader cardiovascular risk [33].

Obesity and MetS are significant mediators in the acne-CVDs link. Patients with acne often exhibit higher BMI, increased waist circumference, and visceral adiposity, all of which are components of MetS. The adipose tissue in obesity acts as an endocrine organ, secreting pro-inflammatory adipokines that perpetuate systemic inflammation [33, 34, 37]. The mTORC1 signaling pathway, central to acne pathogenesis, is also activated in obesity and MetS, promoting lipid accumulation, insulin resistance, and inflammatory responses. Dietary factors, such as high glycemic index foods and dairy consumption, further exacerbate both acne severity and metabolic disturbances, reinforcing the interconnectedness of these conditions [33, 34, 37]. As a result, the recognition of acne as a potential marker for cardiovascular risk necessitates a paradigm shift in clinical management [33, 34]. Dermatologists and primary care providers should adopt a multidisciplinary approach, incorporating cardiovascular risk assessment into routine acne care. Screening for metabolic parameters such as lipid profiles, fasting glucose, insulin resistance, and blood pressure should be considered, particularly in patients with severe or persistent acne.

Lifestyle modifications, including dietary interventions, weight management, and smoking cessation, play a crucial role in mitigating cardiovascular risk in patients with acne [33, 34]. Pharmacological therapies targeting both acne and metabolic dysfunctions, such as metformin, have shown promise in improving insulin sensitivity and reducing inflammatory markers [41]. Additionally, anti-androgen therapies and isotretinoin may influence lipid metabolism, warranting careful cardiovascular monitoring during treatment [42].

Recognizing acne as part of a broader systemic inflammatory spectrum offers opportunities for early cardiovascular risk stratification and preventive interventions, ultimately improving long-term health outcomes in affected individuals.

#### Take Home Message

Acne vulgaris is a systemic inflammatory condition linked to dyslipidemia, insulin resistance, hypertension, and increased cardiovascular risk. Metabolic screening should be integrated into acne management, particularly in high-risk individuals. Targeted interventions, including dietary modifications, weight management, and drugs, may improve both dermatological and cardiometabolic outcomes.

### The Role of Nutrition in Acne

The relationship between nutrition and acne remains a subject of ongoing research. While historically debated, growing evidence suggests that specific dietary components influence sebaceous gland activity, bacterial proliferation, and inflammation—key factors in acne development [43]. High-glycemic index foods, dairy products, chocolate, and saturated fats have been identified as potential aggravators [44, 45]. Additionally, alcohol, processed foods, gluten, and sugary beverages may contribute to acne flares, although further studies are needed to confirm these associations [46, 47].

Acne extends beyond physical manifestations, significantly impacting self-esteem, social interactions, and psychological well-being. Given its substantial effect on quality of life, identifying dietary triggers could serve as an adjunctive strategy in acne management [48]. Clinicians should consider personalized dietary recommendations alongside conventional treatments, encouraging patients to minimize acne-exacerbating foods while promoting a balanced, nutrient-rich diet [49].

Acne vulgaris is a chronic inflammatory condition influenced by multiple factors, including gut microbiota and diet [50, 51]. The Western diet, characterized by high saturated fat and high-glycemic index foods, is linked to acne through dysregulated nutrient signaling, increased sebum production, and *Cutibacterium acnes* overgrowth [52, 53]. A diet rich in fats and sugars may alter gut microbiota diversity, promoting inflammation and intestinal permeability, thereby exacerbating acne symptoms [54]. On the other hand, several dietary patterns have shown potential benefits in acne management. A vegetarian diet, emphasizing plant-based foods while limiting animal products, has been associated with improved acne outcomes [55]. Some studies suggest that eliminating dairy and high-glycemic foods may reduce acne severity by lowering IGF- 1, a key driver of sebum production and inflammation [56]. Vegan diets, which are rich in phytoestrogens and isoflavones, may further support acne reduction by modulating androgen activity and decreasing inflammatory markers [57], though more research is needed to confirm these effects.

Dietary modifications, particularly reducing dairy, processed foods, and high-glycemic foods while adopting plant-based patterns, may serve as supportive strategies for acne management [58]. However, further large-scale, well-controlled studies are necessary to establish definitive dietary guidelines for patients with acne. Until then, integrating dietary adjustments with medical interventions may provide a holistic approach to reducing acne severity and improving patient outcomes [59].

#### Take Home Message

Emerging evidence suggests that diet plays a role in acne development, with high-glycemic foods, dairy, and processed foods acting as potential aggravators. While more research is needed, adopting a nutrient-rich, plant-based or Mediterranean-style diet may help manage acne symptoms by reducing inflammation and sebum production. Personalized dietary recommendations, alongside conventional treatments, could offer a comprehensive approach to improving acne management.

### The Impact of Mediterranean Diet on Acne

Numerous studies have expanded the role of the MedDiet beyond its well-established benefits in cardiovascular diseases to include dermatological diseases. In the context of acne, the MedDiet appears to have a positive impact, influencing both the risk of developing acne and its severity.

A community-based case–control study by Skroza et al. evaluated adherence to the MedDiet using a 10-point MedDiet scale in 293 individuals (93 individuals with acne and 200 healthy controls) [60]. Interestingly, logistic regression analysis showed that familial hypercholesterolemia, type 2 diabetes, and hypertension were strong risk factors for acne (AOR 8.79, 95% CI 1.67–46.22; 3.32, 95% CI 1.27–8.63; and 2.73, 95% CI 1.07–6.96, respectively), while the MedDiet represented a protective factor (score ≥ 6, AOR 0.31, 95% CI 0.11–0.89) [60].

Bertolani et al. conducted a study in 35 normal weight individuals aged 14—30 years affected by acne and treated in line with the European Dermatology Forum (EDF) guidelines [61]. Individuals were divided into 2 groups based on a questionnaire score assessing the adherence to the MedDiet: the Mediterranean Group (score ≥ 6, *n* = 27) and the Western Group (score < 5, *n* = 8). IGF- 1 levels were measured in all individuals before and after treatment and then compared to healthy population. IGF- 1 levels were higher in individuals with acne than in controls and in the Western group than in the Mediterranean group [61]. A case–control study by Ah-Thiane et al., enrolling 40 normal weight women with mild-to-severe acne, and 40 healthy controls, reported a significant negative association between acne severity and adherence to the MedDiet [58]. Further insights were provided by Barrea et al. in a cross-sectional study of 102 individuals (51 with acne, BMI 24.7 ± 4.1 kg/m^2^, and 51 healthy controls, BMI 24.6 ± 1.7 kg/m^2^) [62]. The authors reported worse body composition, including a lower phase angle (PhA) and reduced adherence to the MedDiet, in individuals with acne compared to controls, independent of age, sex, and BMI. Furthermore, both PhA and adherence to the MedDiet were significantly reduced in individuals with severe acne compared to those with mild or moderate forms [62]. Notably, PhA, a simple and accessible parameter derived from bioelectrical impedance analysis, serves as an indicator of cellular integrity and has recently been recognized as a surrogate marker of inflammation, with lower values indicating greater inflammatory status [63]. In contrast, a recent case–control study of 242 university students (71.9% normal weight) found no significant association between adherence to the MedDiet and acne diagnosis or severity [49].

These findings underscore the potential role of the MedDiet in mitigating acne severity through mechanisms likely involving its anti-inflammatory and antioxidant properties [64]. Specifically, the high intake of fruits, vegetables, whole grains, and extra virgin olive oil associated with the MedDiet provides a rich source of polyphenols, vitamins, and omega- 3 fatty acids, which can reduce oxidative stress and modulate inflammatory pathways implicated in acne pathogenesis [64]. Additionally, the low glycemic load of the MedDiet may help regulate IGF- 1, a key driver of sebaceous gland activity and acne development [65].

However, study heterogeneity, small sample sizes, varying dietary assessment tools, and population differences limit generalizability (Table 1). Most studies are observational, precluding causal inferences. Longitudinal, randomized controlled trials are needed to confirm the MedDiet’s benefits in acne management.

**Table 1:** Clinical studies on the Mediterranean Diet and the Very Low-Energy Ketogenic Therapy in individuals with acne

Authors	Year	Study design	Study population	Type of diet	Duration	Severity index assessed	Results	Main conclusions
Taha et al [49]	2024	Case–controlled, cross-sectional study	121 cases (aged 20.3 ± 1.7 years); 121 age, sex and BMI matched healthy controls. Most participants in both groups had a BMI within the healthy range (71.9%, *n* = 174)	MedDiet (MEDAS 10-items questionnarie was used to assess adherence to the MedDiet)	n/a	GAGS	The proportion of participants with high adherence to the MedDiet was slightly higher in controls (22.3%, *n* = 27) compared to individuals with acne (19.8%, *n* = 24). Case–control differences in adherence to the MedDiet and its individual components did not reach statistical significance, except for vegetable consumption (*p* = 0.022). Conditional logistic regression showed that adherence to the MedDiet was not significantly associated with acne diagnosis and severity	Adherence to the MedDiet was not correlated with acne diagnosis or clinical severity. More research on the association between acne and adherence to the MedDiet is needed, as earlier studies are few, were conducted in specific settings, and used variable methodologies
Ah-Thiane et al [58]	2022	Case–controlled, cross-sectional study	40 cases with mild-to-severe acne (aged 19.8 ± 4.3 years; BMI 21.8 ± 3.1 kg/m^2^); 40 age and BMI matched healthy controls	MedDiet (PREDIMED 14-item questionnaire was used to assess adherence to the MedDiet)	n/a	GEA	A global linear model identified a significant negative correlation between the severity of acne and the adherence to the MedDiet in individuals with acne (r = − 0.17; *p* = 0.017). Subgroup analysis revealed a significant positive correlation between consuming more than seven glasses of wine per week and acne severity (*p* = 0.012)	Adoption of a MedDiet could be beneficial to women with moderate-to-severe acne. Contrary to preconceived ideas individuals with acne should be advised to reduce their wine consumption
Barrea et al [62]	2021	Case–controlled, cross-sectional study	51 cases with mild-to-severe acne (aged 23.5 ± 5.9 years; BMI 24.7 ± 4.1 kg/m^2^), treatment naive; 51 age, sex and BMI matched healthy controls	MedDiet (PREDIMED 14-item questionnaire was used to assess adherence to the MedDiet)	n/a	GAGS	Individuals with acne had a worse body composition, with significantly smaller PhA (*p* = 0.003) and lower adherence to the MedDiet (*p* < 0.001) compared to controls. Stratifying acne patients by GAGS categories, both PhA (*p* = 0.006) and PREDIMED score (*p* = 0.007) were significantly lower in those with severe acne compared to those with mild/moderate acne. GAGS score negatively correlated with both PhA (*p* < 0.001) and PREDIMED score (*p* < 0.001). Multivariate analysis showed that PhA and PREDIMED score were the major determinants of the GAGS score (*p* < 0.001). At ROC analysis, the threshold values of PREDIMED score predicting the highest GAGS severity were found at score ≤ 9 (*p* = 0.001, AUC 0.747, standard error 0.076, 95% CI 0.598 to 0.896)	Novel correlations were found between PhA and adherence to the MedDiet with acne severity. Both PhA and PREDIMED scores could potentially serve as markers of acne severity in a clinical setting
Bertolani et al [61]	2021	Case–controlled, cross-sectional study	35 cases with mild-to-severe acne treated according to EDF guidelines (aged 21.0 ± 4.8 years); 13 age matched healthy controls. Normal weight	MedDiet (PREDIMED 14-item questionnaire was used to assess adherence to the MedDiet)	n/a	GAGS	Individuals scoring PREDIMED ≥ 6 points were classified as the MedDiet Group (MD—group), while those scoring < 5 points formed the Western Diet Group (WD-group). IGF- 1 levels were higher in individuals with acne than in controls and in the WD-group than in the MD-group	MedDiet could have a protective role in the pathogenesis of acne by acting on the systemic route of IGF- 1
Skroza et al [60]	2012	Case–controlled, cross-sectional study	93 cases with first-time diagnosed mild-to-severe acne (median age 17 years); 200 age and sex matched healthy controls. BMI not reported	MedDiet (MDS 10-item questionnaire was used to assess adherence to the MedDiet)	n/a	None	MDS score ≥ 6 revealed a protective effect towards acne (crude OR 0.22, 95% CI 0.08–0.64). Logistic regression analysis showed that familial hypercholesterolaemia, diabetes, and hypertension are strong risk factors for acne (AOR 8.79, 95% CI 1.67- 46.22; 3.32, 95% CI 1.27–8.63; and 2.73, 95% CI 1.07–6.96, respectively), while the MedDiet represents a protective factor (MDS score ≥ 6, AOR 0.31, 95% CI 0.11–0.89)	The odds for familial dysmetabolisms were higher in individuals with acne than in controls, confirming their role in determining or maintaining acne
Verde et al [68]	2024	Prospective	31 treatment-naïve women diagnosed with moderate acne and grade I obesity	VLEKT	45 days	GAGS	Significant reduction of GAGS (Δ% = − 31.46 ± 9.53, *p* < 0.001)	45 days of VLEKT improved clinical outcomes in acne, as demonstrated by a significant reduction in GAGS. The observation of the significant reduction in dROMs and TMAO levels after the ketogenic period makes it possible to identify reduction in OxS and improvement in dysbiosis as the two mechanisms underlying the improvement in clinical severity of acne. In this context, improvement in gut dysbiosis is also an additional mechanism for reducing inflammation in acne, contributing to the improvement in disease severity

*BMI* body mass index; *MEDAS* Mediterranean Diet Adherence Screener; *MedDiet* Mediterranean diet; *GAGS* global acne grading system; *PREDIMED* PREvención con DIeta MEDiterránea; *GEA* Global Evaluation Acne; *PhA* phase angle; *ROC* Receiver Operating Characteristic; *AUC* Area Under Curve; *CI* confidence interval; *EDF* European dermatology forum; *MD* Mediterranean diet; *WD* Western Diet, *IGF- 1* insulin-like growth factor- 1; *MDS* Mediterranean Diet Scale; *OR* Odds ratio; *AOR* adjusted odds ratio; *VLEKT* Very-Low Energy Ketogenic Therapy; *dROMs* Reactive Oxygen Metabolites; *TMAO* Trimethylamine N-Oxide, *OxS* Oxidative Stress

#### Take Home Message

The MedDiet shows promise in reducing acne severity, likely due to its anti-inflammatory, antioxidant, and low glycemic properties. While findings suggest benefits, limitations of current research highlight the need for robust, randomized controlled trials to confirm its efficacy.

### The Impact of the Ketogenic Diet on Acne

The first insights into the possible effects of ketosis on acne progression date back over ten years [66, 67]. The connection between ketogenic therapy and acne would find its rationale in the reduction of the glycaemic load of the diet, which, by regulating glycaemic and insulinemic homeostasis, would lead to beneficial effects on skin quality. In particular, the reduction of insulin levels would first of all inhibit the activity of IGF- 1, blocking a series of events involved in the pathogenesis of acne, such as hyperproliferation of keratinocytes in the pilosebaceous ducts, androgen-mediated hyperproduction of sebum, altered desquamation of the follicular epithelium, and proliferation of *Propionibacterium acnes* in the stratum corneum [66, 67]. The potential of ketosis in improving clinical outcomes of acne is also supported by an action in the management of inflammation, which, together with the immune response, is closely implicated in its pathogenesis [13]. In this context, given the marked anti-inflammatory potential of ketogenic therapy, the hypothesis has been speculated that VLEKT may represent a valid strategy for the multifactorial management of acne [13]. To confirm this hypothesis, a recent study by Verde et al. investigated the effect of 45 days active phase of VLEKT in 31 treatment-naïve women diagnosed with moderate acne and grade I obesity (Table 1) [68]. The ketogenic therapy followed the Ketogenic Nutritional Therapy (KeNuT)-SIE protocol [69]. After 45 days of VLEKT, the researchers observed a significant improvement in acne severity index (global acne grading system, GAGS) and quality of life (Dermatology Life Quality Index, DLQI Δ%:—45.44 ± 24.02 score, *p* < 0.001). Similarly, significant reductions in serum levels of derivatives of reactive oxidative metabolites (dROMS, Δ%:—38.07 ± 18.40 U Carr, *p* < 0.001) and trimethylamine N-oxide (TMAO, Δ%:—51.97 ± 15.98 μM, *p* < 0.001) as markers of oxidative stress and intestinal dysbiosis, respectively, were observed. Interestingly, changes in TMAO and dROMs levels were positively correlated with changes in GAGS, underlining that the VLEKT-induced reduction in oxidative stress and improvement in dysbiosis could be the mechanism underlying the improvement in clinical acne outcomes [68].

#### Take Home Message

For the multifactorial management of acne, the available evidence allows us to suggest 45 days of active phase of VLEKT according to the KeNuT-SIE protocol (650—800 kcal/day; < 30 g of carbohydrates *per* day; 20 g of lipids *per* day; 0.8–1.2 g of protein *per* kg of ideal body weight *per* day). This ketogenic therapy allows an improvement in acne management efficacy by acting with antioxidant/anti-inflammatory mechanisms and improving dysbiosis.

**Fig. 1:**
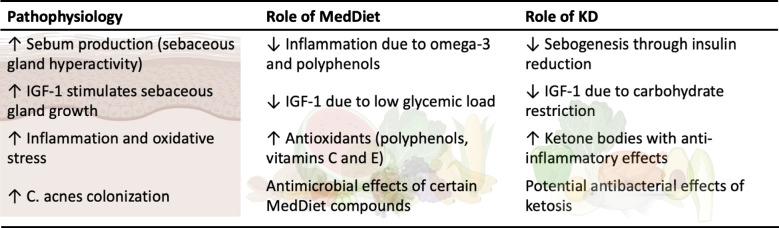
Pathophysiology and role of the MedDiet and ketogenic diet in acne disease, summarizes the pathophysiology and role of the Mediterranean and ketogenic diets in acne disease. *IGF- 1, Insulin-Like Growth Factor 1; C., Cutibacterium; MedDiet, Mediterranean Diet; KD, ketogenic diet*

Figure 1 shows a summary of the pathophysiology and role of the MedDiet and ketogenic diet in acne disease.

## Medical Nutrition Therapy in Hidradenitis Suppurative

### Pathophysiology of Hidradenitis Suppurativa

HS is an inflammatory dermatological disease presenting with nodules, abscesses and tunnels on apocrine sweat gland-bearing skin [70], whose pathogenesis is complex and multifactorial involving genetic/epigenetic, hormonal and environmental factors such as obesity and smoking, immune dysregulation, and skin microbiome alterations [6, 71]. It has recently been suggested that HS may be regarded as an autoinflammatory keratinization disease due to causative genes involved in keratinization and autoinflammation pathways [72, 73], the latter specifically belonging to the Th1/Th17 axis [74]. In addition, although still poorly explored in HS, evidence for a shift in thiol/disulfide balance toward disulfides and an increased ischemia-modified albumin, revealed a potential relationship of oxidative status in HS inflammatory context [75]. More recently, a shift in cysteine-related metabolism has also been reported, implying increased cystine and taurine levels that integrate with a depletion of glutathione, docosahexaenoic acid metabolites [76], and dihydroxyeicosatrienoic acids mediators, reinforcing the damaging role of ROS in HS chronic inflammation [77].

Oxidative stress can also be induced by altered aryl hydrocarbon receptor signaling and diet. Indeed, a diet particularly high in carbohydrates as well as a deficiency of zinc and vitamin B12 have been associated to neutrophil function and increased levels of ROS and inflammatory cytokines such as TNF-α, IL- 1β, IL- 6 and IL- 18 [78]. Consistently, unhealthy diet may favour obesity, a well-known risk factor for HS, in which there is evidence for increased levels of insulin and insulin-like growth factor that suppress the mTOR pathway and activate androgen receptors, resulting in cell hyperproliferation within the pilosebaceous unit and secretion of inflammatory mediators [79]. At the same time, obesity leads to insulin resistance and favours oxidative stress-related mechanisms, mechanical stress and follicular hyperkeratosis [80], negatively influencing HS burden and activity [80].

#### Take Home Message

HS is an autoinflammatory dermatological disease, in which a close interaction between inflammatory and oxidative processes contributes to its pathogenesis. A holistic approach to HS management, also considering the role of dietary factors may improve disease activity, ameliorating patient’s quality of life.

### The Role of Hormones in Hidradenitis Suppurativa

Endocrine involvement in the pathogenesis of HS is supported by experimental and clinical evidence. Sex hormones and prolactin may participate in the development and progression of HS by influencing skin homeostasis and contributing to infundibular hyperkeratinization and occlusion of hair follicles [81]. HS lesions showed increased expression of androgen receptors in the infundibulum and skin tunnel compared to healthy skin, and their expression was higher in males than in females [82]. Indeed, female patients with HS have an elevated testosterone and free androgen index. Clinical support is also provided by peripubertal onset of HS, high prevalence of acne history, and greater risk of polycystic ovary syndrome. In addition, there is a clear gender difference in the clinical presentation of the disease. The severity of HS changes along the women reproductive life, as HS exhibits a common peri‑menstruation flare‑up in its activity, linked to the progressive increase in progesterone levels, while HS usually improves after menopause and hormone-related therapy. Conflicting effects are often described during pregnancy, but postpartum exacerbation is commonly reported, possibly associated with increase in prolactin levels. Progestogen-only pill worsened the HS symptoms, while beneficial effects are exerted for female patients by combined oral contraceptive pills, and anti-androgen agents, such as cyproterone acetate, a competitive antagonist at the androgen receptor, finasteride, an inhibitor of type 5α-reductase, the enzyme converting testosterone and its active metabolite 5α-dihydrotestosterone, or spironolactone, a potassium-sparing diuretic acting as competitive aldosterone receptor antagonist and exerting weak anti-androgenic effects [82]. Of interest, many patients with HS have MetS, impaired glucose tolerance and insulin resistance, with a 1.69-fold increased risk of developing type 2 diabetes [83], and there are positive reports regarding metformin, a diabetic drug increasing insulin sensitivity with mild anti-androgen effects, and weight loss after diet and bariatric surgery [82]. Recent findings indicate that glucagon-like peptide- 1 receptor agonists, widely known for lowering blood glucose levels and promoting weight reduction, may also offer therapeutic benefits in managing HS [84].

#### Take Home Message

There is consolidate evidence that sex hormones and altered glucose metabolism play an important role in HS, providing also an important starting point for further research in HS pathogenesis and management.

### Hidradenitis Suppurativa and Obesity

HS is strongly associated with obesity, a condition characterized by chronic low-grade inflammation [85]. Research has revealed that obesity may play a pivotal role in HS pathogenesis through its influence on systemic inflammation and metabolic dysfunction [86]. Obesity, frequently observed in individuals with HS, is accompanied by an altered production of adipokines, such as resistin and chemerin, as well as elevated inflammatory cytokines IL- 1β and TNF-α, which contribute to inflammatory processes and metabolic derangements [86].

A review by Krajewski et al. noted that individuals with HS exhibit an imbalance in adipokine levels, with increased proinflammatory adipokines (leptin, resistin, and visfatin) and decreased anti-inflammatory adiponectin [87]. These changes promote systemic inflammation and may directly contribute to HS development [87]. This adipokine dysregulation is consistent with findings from González-López and colleagues, reporting lower serum adiponectin levels and higher leptin, resistin, and visfatin levels in patients with HS compared to healthy controls [88]. These differences persisted even after adjusting for age, sex, and BMI, indicating that these adipokine alterations are intrinsic to HS pathology rather than merely a consequence of obesity. Moreover, resistin and visfatin were identified as independent risk factors for HS, while adiponectin levels were inversely correlated with insulin resistance, a condition prevalent among individuals with HS [88].

It is evident that a central mechanism linking HS and obesity is represented by the state of chronic inflammation, known as meta-inflammation [85]. Mintoff et al. emphasized that lifestyle and genetic factors, combined with obesity-driven meta-inflammation, exacerbate HS severity. Targeting such inflammatory noxa through weight management or modulation of adipokine levels may offer novel therapeutic approaches for HS [85].

In conclusion, the link between obesity and HS is mediated by systemic inflammation and dysregulated adipokine profiles. Reduced adiponectin and elevated resistin and visfatin levels not only contribute to HS pathogenesis but may also serve as biomarkers for disease risk and progression. These findings underscore the importance of addressing obesity and associated metabolic abnormalities as part of comprehensive HS management.

#### Take Home Message

Obesity plays a key role in HS pathogenesis through systemic inflammation and dysregulated adipokine levels. Elevated proinflammatory adipokines (resistin, visfatin) and reduced adiponectin contribute to HS severity, independent of BMI. Targeting obesity-driven meta-inflammation through weight management and modulation of adipokines may offer promising therapeutic strategies.

### Hidradenitis Suppurativa and Cardiovascular Risk

HS is increasingly recognized not only as a chronic inflammatory skin disorder but also as a systemic condition with significant cardiovascular implications [89]. The association between HS and CVDs is multifaceted, involving shared inflammatory pathways, metabolic dysregulation, and behavioral risk factors such as obesity and smoking [89]. Chronic systemic inflammation, a hallmark of HS, plays a pivotal role in the development and progression of atherosclerosis, hypertension, and other cardiovascular conditions, positioning HS as a potential independent risk factor for CVDs [18, 90, 91].

Hypertension is one of the most prevalent cardiovascular comorbidities in patients with HS [91]. Several studies have demonstrated a significantly higher prevalence of hypertension among individuals with HS compared to the general population. In six out of eight studies examining this relationship, the aOR for hypertension ranged from 1.2 to 2.1, indicating a moderate but consistent increase in risk [91]. The prevalence of hypertension in patients with HS varied widely, from 7.8% to 56.3%, reflecting differences in study populations and diagnostic criteria. Interestingly, one study reported a lower risk of hypertension (OR 0.7) in patients with HS compared to controls; however, this finding may be limited by the study’s focus on single inpatient admissions without accounting for prior comorbidities [91, 92].

The pathophysiological mechanisms linking HS to hypertension are complex and likely multifactorial [91]. Chronic systemic inflammation, characterized by elevated levels of pro-inflammatory cytokines such as TNF-α and IL- 6, contributes to endothelial dysfunction, increased arterial stiffness, and dysregulation of the renin–angiotensin–aldosterone system, all of which are key factors in the development of hypertension [90, 91, 93]. Additionally, behavioral risk factors prevalent in HS populations, including high rates of tobacco use and obesity, further exacerbate the risk of hypertension [91, 92].

HS is also associated with an increased risk of major adverse cardiovascular events (MACE), which include myocardial infarction, cerebrovascular accidents (stroke), and cardiovascular-related mortality [89]. In a large retrospective population-based cohort study, the incidence rates of MACE were 40 *per* 10.000 person-years in patients with HS compared to 19 *per* 10.000 person-years in healthy control individuals. After adjusting for traditional cardiovascular risk factors, the incident risk of MACE remained significantly elevated in patients with HS, with an aOR of 1.5 (95% CI, 1.27–1.86). Moreover, the risk of cardiovascular death was 1.6 times higher in patients with HS compared to those with psoriasis, a condition already known to be associated with increased cardiovascular risk [89].

This elevated risk is thought to be driven by persistent systemic inflammation, which promotes atherogenesis through endothelial injury, lipid oxidation, and plaque formation [36, 89]. Elevated circulating levels of TNF-α and IL- 6 in patients with HS contribute to a pro-inflammatory, pro-thrombotic state, increasing the likelihood of acute cardiovascular events [90, 93]. Additionally, HS-associated metabolic abnormalities such as insulin resistance, dyslipidemia, and obesity amplify the risk of MACE [91, 92].

Emerging evidence suggests that HS is linked to subclinical atherosclerosis, even in the absence of overt cardiovascular disease [36, 94]. Subclinical atherosclerosis can be assessed using surrogate markers such as carotid intima-media thickness (CIMT) and the presence of carotid plaques [36, 94]. In a single-center study, patients with HS exhibited significantly greater mean CIMT and a higher prevalence of carotid plaques compared to age- and sex-matched controls [94]. The risk of carotid plaque formation was three times higher in patients with HS (aOR 3.0; 95% CI, 1.26–7.13), highlighting the early vascular changes that may precede clinical cardiovascular events [95].

A prospective observational study further demonstrated that subclinical atherosclerosis was present in 30.6% of patients with HS compared to 16.1% of control individuals [96]. Among patients with HS over the age of 40, the odds of having subclinical atherosclerosis were three times greater than in controls (95% CI, 1.0–9.5). These findings underscore the importance of early cardiovascular risk assessment in patients with HS, even in those without traditional risk factors [96].

Chronic systemic inflammation is the central pathogenic mechanism connecting HS to cardiovascular disease. HS is characterized by dysregulation of the innate and adaptive immune systems, leading to persistent inflammation both in the skin and systemically [90, 93]. Elevated levels of inflammatory cytokines such as TNF-α, IL- 1β, IL- 6, and C-reactive protein (CRP) are commonly observed in patients with HS [90, 93]. These inflammatory mediators contribute to endothelial dysfunction, oxidative stress, and vascular remodeling, all of which are key processes in the development of hypertension, atherosclerosis, and thrombosis [36, 90].

Furthermore, systemic inflammation in HS promotes insulin resistance, dyslipidemia, and hypercoagulability, creating a pro-atherogenic environment [36]. The role of TNF-α is particularly notable, as it not only drives skin inflammation in HS but also contributes to vascular inflammation, plaque instability, and increased risk of acute cardiovascular events [36].

Given the strong association between HS and CVD, routine cardiovascular risk assessment is essential in the management of patients with HS [91, 92, 96]. Screening for hypertension, dyslipidemia, type 2 diabetes, and other components of MetS should be integrated into standard care protocols. Additionally, non-traditional risk markers, such as CIMT and carotid plaque assessment, may help identify subclinical atherosclerosis in high-risk patients [91, 92].

#### Take Home Message

HS is not just a skin disorder but a systemic condition with significant cardiovascular implications. Chronic systemic inflammation in HS contributes to hypertension, atherosclerosis, and an increased risk of MACE. Elevated pro-inflammatory cytokines and metabolic abnormalities drive vascular dysfunction and cardiovascular risk. Routine cardiovascular risk assessment, including screening for hypertension, dyslipidemia, and subclinical atherosclerosis, is essential for comprehensive HS management.

### The Role of Nutrition in Hidradenitis Suppurativa

In recent years, diet has been evaluated as a potential factor in the pathogenesis and treatment of HS [97]. Population-based studies have shown that the incidence of HS is significantly higher in Westernized societies, suggesting a role for dietary habits in disease development [97]. Despite the growing interest, the current evidence remains limited, with most studies focusing on weight loss diets, exclusion diets, and micronutrient supplementation [97].

A direct association between BMI and disease severity has been observed [98], highlighting the potential benefits of dietary interventions. Weight-loss therapies, particularly the MedDiet, the ketogenic diets, and the carbohydrate-reduced diets, have shown promise in reducing disease flare-ups [97]. The latter two are particularly relevant due to the high prevalence of hyperinsulinemia in individuals with obesity and HS, as well as the pro-inflammatory effects of high-glycemic-load diets [97]. Beyond weight loss, micronutrient deficiencies have been implicated in HS pathogenesis [99]. Patients with HS are at an increased risk of deficiencies in iron, zinc, vitamin D, and vitamin B12. Supplementation with zinc, vitamin D, vitamin B12, and vitamin A has been shown to have beneficial effects in patients with HS [99]. Additionally, exclusion diets have been explored to assess their role in disease management [97]. Studies have investigated the impact of eliminating milk and dairy products, given their role in increasing IGF- 1 levels [100], and the exclusion of brewer's yeast, which has been reported as a potential trigger for HS exacerbations [101]. However, improvements observed in these cases may also be attributed to the adoption of an overall healthier diet.

Furthermore, diet can influence HS pathogenesis through modulation of the gut microbiome [102]. Intestinal dysbiosis, often associated with high-fat diets, has been proposed as a contributing factor to HS by influencing skin microbiota and systemic inflammatory pathways. Probiotics have emerged as a potential therapeutic option for restoring microbial balance and improving skin health in patients with HS [102].

#### Take Home Message

Dietary patterns play a crucial role in the pathogenesis and management of HS. Obesity, hyperinsulinemia, and micronutrient deficiencies are key factors influencing disease severity. Weight-loss interventions, exclusion diets, and micronutrient supplementation may provide benefits, although further research is needed to establish definitive dietary guidelines. The gut microbiome represents an emerging area of interest, with probiotics offering a potential therapeutic approach.

### Impact of Mediterranean Diet on Hidradenitis Suppurativa

Evidence consistently indicates that higher adherence to the MedDiet is associated with reduced severity of HS. In a case–control study, Barrea et al. found that individuals with HS (*n* = 41, BMI 31.1 ± 7.7 kg/m^2^) had lower adherence to the MedDiet compared to healthy controls (*n* = 41, BMI 30.9 ± 7.0 kg/m^2^) [103]. A PREDIMED score ≤ 5.0 was identified as a marker for greater disease severity, with adherence to the MedDiet explaining 30.4% of the variability in Sartorius scores [103]. Similarly, Velluzzi et al. confirmed low adherence to the MedDiet in individuals with HS (n = 35, BMI 27.3 ± 6.3 kg/m^2^) compared to age- and sex-matched healthy controls (BMI 23.1 ± 2.8 kg/m^2^) in a case–control study [104]. Lorite-Fuentes et al. also demonstrated that higher adherence to the MedDiet was associated with lower disease severity, as assessed by International Hidradenitis Suppurativa Severity Score System (HIS4) and Hurley scores, in a cohort of 221 individuals with HS (BMI 29.7 ± 6.3 kg/m^2^) [105]. Notably, the use of extra virgin olive oil was linked to reduced disease activity [105]. More recently, Kesik et al. reinforced these findings in a case–control study involving 1004 individuals with HS (BMI 23.6–29.8 kg/m^2^) and 4436 healthy controls (BMI 23.1–28.1 kg/m^2^) [106]. Their study revealed significant negative correlations between adherence to the MedDiet and both IHS4 and Hurley stages, with the MedDiet emerging as a key variable influencing disease severity [106]. Finally, Bouwman et al. further supported these results by showing that lower MedDiet adherence increased the risk of HS severity [107]. Their study also demonstrated a 1.07-fold increased risk of HS severity for every standard unit decrease in adherence to the MedDiet, based on data from individuals who developed HS during follow-up [107]. These studies collectively underscore the role of the MedDiet as a potential modifiable factor in mitigating the severity of HS.

The MedDiet may improve HS severity through multiple mechanisms, including promoting weight loss, which is closely linked to reduced disease activity, and mitigating hyperinsulinemia and elevated IGF- 1 levels, both of which exacerbate androgen-driven follicular obstruction [108, 109]. Additionally, the MedDiet’s low glycemic load, high content of anti-inflammatory and antioxidant compounds from fruits, vegetables, and olive oil, and limited dairy consumption align with dietary modifications shown to alleviate HS symptoms and reduce disease progression [108, 109].

While promising, studies on individuals with HS are largely cross-sectional (Table 2), limiting causal inference. High-quality randomized controlled trials are necessary to confirm these findings and to establish specific dietary guidelines for individuals with HS.

**Table 2:** Clinical studies on the Mediterranean Diet and the Very Low-Energy Ketogenic Therapy in individuals with Hidradenitis Suppurativa

Authors	Year	Study design	Study population	Type of diet	Duration	Severity index assessed	Results	Main conclusions
Kesik et al [106]	2024	Case–controlled, cross-sectional study	50 cases with 1-to- 3 stage HS (aged 34.1 ± 11.4 years; BMI 28.0 ± 6.1 kg/m^2^); 40 age, sex and BMI matched healthy controls	MedDiet (MEDAS 10-items questionnaire was used to assess adherence to the MedDiet)	n/a	IHS4 and Hurley Stages	Individuals with HS exhibited significantly lower adherence to the MedDiet compared to controls (*p* < 0.001). The MEDAS scores were negatively correlated with the IHS4 scores (*p* < 0.001). The Hurley stage was negatively correlated with the MEDAS scores (*p* < 0.001). In multiple regression analysis, the MEDAS score emerged as the primary variable associated with disease severity (*p* < 0.001)	These findings suggest that an increased adherence to the MedDiet positively affect HS management
Bouwman et al [107]	2024	Case–controlled, cross-sectional study	1004 cases with 1-to- 3 stage HS (aged 55.2 ± 12.3 years; BMI 26.1 [23.6–29.8] kg/m^2^); 4436 age matched healthy controls (BMI 25.3 [23.1–28.1] kg/m^2^)	MedDiet (aMED 8-items questionnarie was used to assess adherence to the MedDiet)	n/a	Hurley Stages	Individuals with HS had lower adherence to the MedDiet compared to controls (*p* < 0.001), resulting in a significantly increased risk of 1.07 [1.12–1.03] *per* 1-standard unit increase in aMED (*p* = 0.01).'HS developers'(those who developed HS during follow-up) also exhibited a trend of lower aMED compared to controls, leading to a 1.06 [1.13–0.98] increased risk of HS onset per 1-standard unit increases in adherence to the MedDiet (*p* = 0.04)	Low adherence to the MedDiet was associated with an increased risk of HS in the general population
Lorite-Fuentes et al [105]	2022	Cross-sectional study	221 participants with 1-to- 3 stage HS (aged 38.4 ± 10.7 years; BMI 29.7 ± 6.3 kg/m^2^)	MedDiet (PREDIMED 14-item questionnaire was used to assess adherence to the MedDiet)	n/a	IHS4, Hurley Stages and self-reported disease activity NRS	Higher adherence to the MedDiet was associated with lower IHS4 (*p* < 0.001), lower Hurley Stage (*p* = 0.01) and lower self-reported NRS (*p* = 0.02). The use of EVOO as the main culinary lipid and poultry rather than red meat consumption were the dietary habits that implied a lower degree of disease activity (*p* < 0.05)	Higher adherence to the MedDiet was associated with lower HS severity, suggesting that the MedDiet, due to its anti-inflammatory properties, could be an appropriate dietary pattern for individuals with HS
Velluzzi et al [104]	2021	Case–controlled, cross-sectional study	35 cases with 1-to- 3 stage HS (aged 30.1 ± 10.0 years; BMI 27.3 ± 6.3 kg/m^2^); 41 age and sex matched healthy controls (BMI 23.1 ± 2.8 kg/m^2^)	MedDiet (MedDietScore 12-items questionnarie was used to assess adherence to the MedDiet)	n/a	Hurley Stages and Sartorius score	Adherence to the MedDiet was lower in individuals with HS compared to controls (*p* = 0.007). None of the evaluated variables were correlated with the severity of the disease	Although nutritional factors and lifestyle can be important and modifiable factors in the HS course, the detrimental effect of chronic inflammation and delayed management are clearly prevalent and heavily influence the disease burden
Barrea et al. [111]	2018	Case–controlled, cross-sectional study	41 cases with 1-to- 3 stage HS (aged 26.2 ± 9.9 years; BMI 31.1 ± 7.7 kg/m^2^), treatment naive; 41 age, sex and BMI matched healthy controls	MedDiet (PREDIMED 14-item questionnaire was used to assess adherence to the MedDiet)	n/a	Sartorius score, Hurley Stages, and HS-PGA	Individuals with HS had lower adherence to the MedDiet compared to controls, despite no differences in energy intake between the groups. At ROC analysis, a score of PREDIMED score of ≤ 5.0 (*p* < 0.001, AUC 0.762, standard error 0.077, 95% CI 0.603 to 0.881) could serve as a threshold for a significantly increased risk of high Sartorius scores. After adjusting for sex, age, BMI, and total energy intake, the Sartorius score showed a negative correlation with PREDIMED score (*p* < 0.001). The multivariate analysis revealed that PREDIMED score was a significant determinant of the Sartorius score, explaining 30.4% of its variability (*p* < 0.001)	Adherence to MedDiet might represent possible marker of severity of HS in a clinical setting
Verde et al [14]	2024	Prospective	12 with HS and overweight or obesity (BMI 27.03 to 50.14 kg/m^2^), aged 21 to 54 years	VLEKT	28 days	Sartorius HS score	Significant reduction of Sartorius HS score (Δ% = − 24.37 ± 16.64, *p* < 0.001)	28 days of VLEKT significantly improved the clinical severity of HS in women with obesity, through the reduction of markers of OxS (oxLDL and dROMs) and intestinal dysbiosis (TMAO) These observations are corroborated by the existence of a positive correlation between changes in the Sartorius score and changes in oxLDL, dROMs and TMAO, suggesting reduction of inflammation/OxS and improvement of intestinal dysbiosis, both induced by VLEKT, as the main mechanisms associated with improved clinical outcomes of HS

*HS* Hidadenitis suppurativa; *BMI* body mass index; *MedDiet* Mediterranean diet; *IHS4* International Hidradenitis Suppurativa severity score system; *MEDAS* Mediterranean Diet Adherence Screener; *aMED* alternate Mediterranean diet; *PREDIMED* PREvención con DIeta MEDiterránea; *NRS* Numeric Rating Scale; *EVOO* extra virgin olive oil; *PGA* Physician’s Global Assessment; *ROC* Receiver Operating Characteristic; *AUC* Area Under Curve; *CI* confidence interval; *VLEKT* Very-Low Energy Ketogenic Therapy; *OxS* Oxidative Stress; *ox-LDL* oxidised Low-Density Lipoprotein; *ROMs* Reactive Oxygen metabolites; *TMAO* Trimethylamine N-Oxide

#### Take Home Message

Higher adherence to the MedDiet is associated with reduced HS severity through mechanisms such as mitigation of hyperinsulinemia and IGF- 1 levels, and the anti-inflammatory effects of its components, highlighting the need for randomized controlled trials to confirm its therapeutic potential.

### Impact of the Ketogenic Diet on Hidradenitis Suppurativa

The anti-inflammatory and antioxidant potential of VLEKT forms the basis of the rationale for its use in HS. The first observations on the benefits of ketosis in this specific clinical setting came from a case study reporting the efficacy of the ketogenic diet (not VLEKT) on improving the clinical outcomes of a woman with vaginal HS [110]. More recent evidence, on the other hand, specifically shows that ketogenic therapy can be a valuable support as an MNT in women with HS and overweight or obesity [14] (Table 2). In this case, the active phase of VLEKT elaborated according to the KeNuT-SIE protocol [69] with replacement meals was prescribed for 28 days, at the end of which improvements in anthropometric measurements, body composition and metabolic parameters (reduction of total and LDL-cholesterol) were observed [14]. Similarly, there was both a significant reduction in the Sartorius score (from 56.25 ± 14.27 to 42.83 ± 15.29 score, *p* < 0.001), and a significant increase in PhA (from 5.93° ± 0.77 to 6.83° ± 0.80, *p* < 0.001), a raw parameter of bioelectrical impedance analysis considered a surrogate marker of inflammation [63], showing an inverse relationship with serum levels of biomarkers of inflammation [14]. The improvement in inflammatory status was corroborated by the observation of significant effects of VLEKT on markers of oxidative stress and dysbiosis. In particular, following the 28 days of ketogenic therapy, the authors observed significant reductions in blood levels of (i) oxidised LDL (- 25.53 ± 7.64 μg/mL, *p* < 0.001) and dROMs (- 21.10 ± 12.86 U Carr, *p* = 0.001) as markers of oxidative stress and (ii) TMAO (- 23.67 ± 13.26 μM, *p* < 0.001) [14], a gut-derived metabolite, indicator of dysbiosis and associated with inflammation, already shown to be increased in patients with HS and associated with the clinical severity of the disease [111].

#### Take Home Message

Although the available evidence is scarce, with only one pilot study currently published, the results clearly demonstrate how ketogenic therapy developed according to the KeNuT-SIE protocol (VLEKT) can be a valid strategy for the multifactorial management of HS. In particular, the potential of VLEKT lies primarily in its anti-inflammatory and antioxidant effects. Particularly interesting is its effect in improving intestinal dysbiosis. These, taken together, represent the main putative mechanisms for the improvement of the clinical outcomes of HS promoted by VLEKT.

**Fig. 2:**
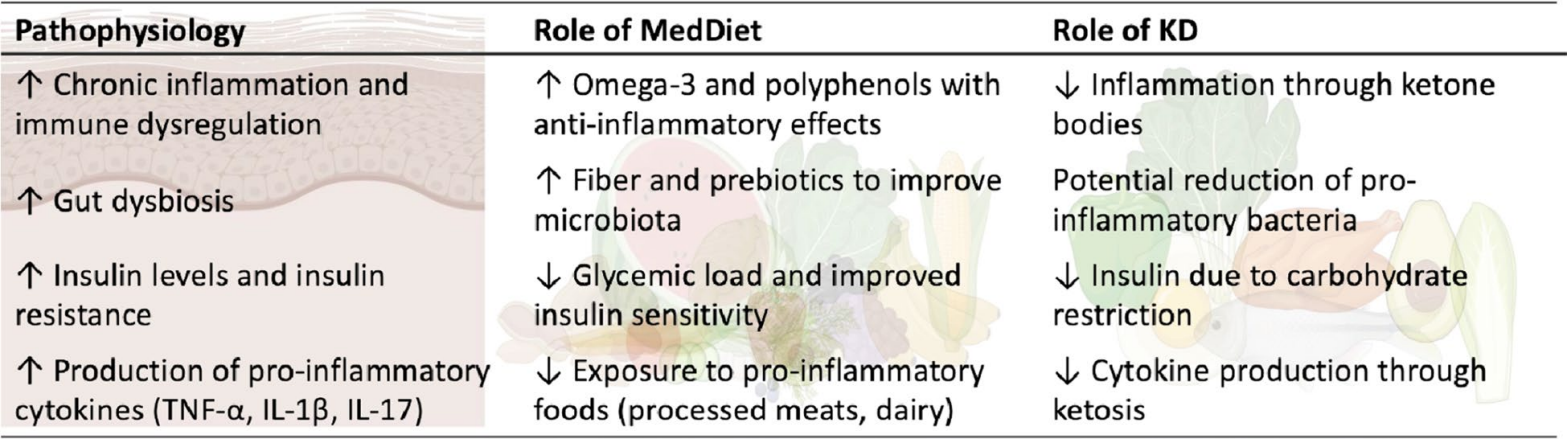
Pathophysiology and role of the MedDiet and ketogenic diet in HS disease, summarizes the pathophysiology and role of the Mediterranean and ketogenic diets in HS disease. *TNF-α, Tumor Necrosis Factor Alpha; IL, Interleukin; MedDiet, Mediterranean Diet; KD, ketogenic diet*

Figure 2 shows a summary of the pathophysiology and role of the MedDiet and ketogenic diet in HS disease.

## Medical Nutrition Therapy in Psoriasis

### Pathophysiology of Psoriasis

Psoriasis is a chronic, inflammatory skin disease with heterogenous cutaneous, articular and systemic manifestations. Environmental factors (including smoking habits, streptococcal infections, and trauma), genetic predisposition (involving major loci as PSORS1 or SNPs in IL23R) and immune mediated pathways contribute to psoriasis pathogenesis and associated comorbidities. The role of TNFα and IL- 23/Th17 pathways is well established [5, 112]. Abnormally increased production of ROS, such as superoxide radical, nitric oxide, malondialdehyde, and inducible nitric oxide synthase, and redox imbalance in antioxidant pathways, including superoxide dismutase, and glutathione peroxidase and enzymes catalase, have been demonstrated not only in individuals with psoriasis but also in metabolic comorbidities and obesity pathogenesis [113, 114]. Many factors including mitochondrial and endothelial dysfunction, hyperglycemia, dyslipidemia, unhealthy diet might generate oxidative stress in individuals with obesity, leading to direct effects on adipocytes maturation and differentiation as well as on hypothalamic center of satiety and huger behaviour [115, 116]. These data suggest that obesity contributes to biochemical mechanisms involved in oxidative stress regulation in patients with psoriasis and that normal weighted patients with psoriasis are at higher risk of obesity due to psoriasis-linked redox imbalance [115, 116]. Dysregulation of ROS can promote abnormal keratinocyte proliferation and differentiation, leading to epidermal hyperplasia and psoriatic plaque formation through MAPK/AP- 1, NF-κB, and JAK/TYK/STAT signaling pathways modulation [117, 118]. Thus, ROS directly influence psoriatic cytokine milieu including TNFα, IL- 22, and IL- 23/Th17 axis molecules and concomitant neutrophils- and monocytes-derived myeloperoxidase promotes endothelial dysfunction and inflammation [119]. Therapeutic modulation of oxidative stress through diet, food supplements and systemic agents including biologics (such as anti-TNFα and anti-IL- 17 agents) and dimethyl fumarate has been reported, confirming the pivotal role of oxidative stress in psoriasis and psoriatic comorbidities pathogenesis [120, 121].

#### Take Home Message

Psoriasis is a multifactorial disease in which inflammatory and oxidative processes play a relevant role not only in cutaneous manifestations but also in comorbidities development. A multidimensional approach to psoriasis treatment, considering the role of diet and obesity improve optimal and stable therapeutic success.

### The Role of Hormones in Psoriasis

The possible involvement of sex steroid in psoriasis pathogenesis is supported by the sex differences in both prevalence and severity of the diseases [122]. Although there is still conflicting evidence, some clinical studies indicate that the prevalence and severity of psoriasis are higher in men than in women, especially during the fertile life of women, while others reported a similar prevalence, but a lower severity in women than men [122].

Experimental studies suggest that estradiol (E2) might exert both inhibitory or facilitating effects in psoriatic inflammation through ERα and ERβ in neutrophils and macrophages, in a context-dependent manner [123]. In particular, E2 play anti-psoriatic functions by down-regulating IL- 1β production from neutrophils monocytes/macrophages through ERα/β, but E2 exerts also facilitating role on psoriatic inflammation by inducing IL- 23 production from dendritic cells through ERα. Although there is general agreement that low E2 levels are associated with a predominant Th1-cell immune responses and pro-inflammatory cytokines, while high E2 levels up-regulate Th2 cell-dependent cytokines, the molecular mechanisms that switch the functions of E2 from pro-inflammatory to anti-inflammatory in psoriasis requires further investigations [123].

In women, the severity of psoriasis changes along each phase of a woman’s life cycle and with a peak of prevalence during puberty and menopause, while the increased production of E2 and progesterone during the menstrual cycle exert anti-inflammatory effects [124]. Pregnancy, along with high E2 levels and increased E2 to progesterone-ratios, correlates with improvement of psoriasis in up to 50% of patients, while in the postpartum period, approximately 65% of patients with psoriasis report a flare up of psoriasis, which correlates also with the high levels of prolactin. However, despite the clinical evidence that serum E2 levels are inversely correlated with psoriasis severity, and replacement therapy with low-dose E2 administration in may improve psoriasis, tamoxifen, an antiestrogen agent, results in the remission of psoriasis, thus suggesting that estrogens might exert both pro-inflammatory and anti-inflammatory effects in psoriasis [124]. However, emerging evidence suggests a complex bidirectional relationship between psoriasis and metabolic disorders, such as obesity, insulin resistance, and type 2 diabetes, highlighting their possible common etiopathogenetic mechanisms [125]. In particular, the prevalence of psoriasis is increased in individuals with obesity, and obesity may aggravate existing psoriasis, while diet, bariatric surgery, and glucagon-like peptide- 1 receptor agonists, a class of drugs for the treatment of type 2 diabetes and obesity, appear to contribute to improving psoriasis lesions [125].

#### Take Home Message

A deficiency of female sex hormones is among the risk factor of psoriasis in women. The immunoregulatory mechanisms of E2 in psoriasis suggest that an appropriate activation of estrogen receptor-signaling is a potential novel therapeutic target in psoriasis, especially in women. Diet and weight loss contribute to improving psoriasis lesions.

### Psoriasis and Obesity

Psoriasis, a chronic systemic inflammatory disease, shares key pro-inflammatory mechanisms with obesity, including cytokines such as interferon-γ, interleukins, and TNF-α [126]. Adipokines further contribute to obesity-driven inflammation in psoriasis, exacerbating psoriasis severity [127]. Obesity is an independent risk factor for psoriasis, influencing disease progression and response to therapies [128].

Epidemiological studies reinforce the connection between obesity and psoriasis. For instance, the Nurses’ Health Study II, which followed 78.626 women, found a positive association between BMI and incident psoriasis [129]. Similarly, the HUNT study in Norway demonstrated that higher abdominal fat and weight gain significantly increased psoriasis risk, while weight reduction was associated with a reduced risk [130].

The Korean Nationwide Cohort Study aimed to evaluate the association between obesity and metabolic status and the incidence of psoriasis [131]. A total of 418.057 participants were stratified according to BMI categories and metabolic status. At the follow-up visit, 11.054 cases (2.6%) were found to have psoriasis. Type 2 diabetes, hypertension, hyperlipidemia and obesity were found to be risk factors for psoriasis. Metabolically unhealthy non-obese subjects (MUNO) (HR 1.29) and metabolically unhealthy subjects with obesity (MUO; HR 1.33) had a significantly higher risk of psoriasis incidence than MUNO [28]. The risk of developing psoriasis was elevated among MUNO and MUO subjects in both sexes and in all age groups. In conclusion, metabolic health was significantly associated with an increased risk of psoriasis in both non-obesity subjects and subjects with obesity [131].

In conclusion, obesity and psoriasis share a bidirectional relationship, with obesity increasing both the risk and severity of psoriasis. This underscores the importance of weight management as a cornerstone of both prevention and treatment strategies for psoriasis.

#### Take Home Message

Obesity and psoriasis are closely linked through shared inflammatory pathways and metabolic dysregulation. Weight management, including lifestyle modifications and metabolic health optimization, should be considered a key component in both the prevention and management of psoriasis.

### Psoriasis and Cardiovascular Risk

Psoriasis is increasingly recognized not merely as a chronic inflammatory skin disorder but as a systemic disease with significant implications for cardiovascular health [132, 133]. The association between psoriasis and cardiovascular risk is complex, involving both traditional risk factors and unique pathogenic mechanisms related to systemic inflammation [132, 133].

Psoriasis affects approximately 2% of the global population and is characterized by hyperproliferation of keratinocytes and immune dysregulation, particularly involving Th1, Th17, and Th22 cells, along with elevated levels of pro-inflammatory cytokines such as TNF-α, IL- 17, and IL- 23 [132, 133]. This chronic inflammatory state is not confined to the skin but also contributes to endothelial dysfunction, atherosclerosis, and an increased prevalence of CVDs, including myocardial infarction, coronary artery disease, and heart failure [133, 134].

Epidemiological studies have consistently demonstrated that patients with psoriasis have a higher prevalence of traditional cardiovascular risk factors, including obesity, hypertension, dyslipidemia, type 2 diabetes, and MetS [133]. Obesity and MetS are particularly prevalent, with studies indicating that up to 30.3% of patients with psoriasis meet the criteria for MetS, significantly higher than in non-psoriatic controls [133, 135]. Psoriasis-related inflammation may exacerbate these conditions through mechanisms such as insulin resistance and lipid metabolism disturbances, further compounding cardiovascular risk [133, 135].

Moreover, psoriasis itself appears to be an independent risk factor for cardiovascular events. The systemic inflammation characteristic of psoriasis promotes atherosclerosis through endothelial activation, increased oxidative stress, and a pro-thrombotic state [135]. A landmark study highlighted that the risk of myocardial infarction (MI) is significantly elevated in patients with psoriasis, particularly in those with severe disease, independent of traditional risk factors [135]. This was corroborated by meta-analyses showing that psoriasis is associated with increased risks of coronary artery disease (RR = 1.51 in Europeans and 1.91 in East Asians) and MI (RR = 1.23 in Europeans and 2.17 in East Asians), while the association with heart failure remains less clear [133, 134]. The pathogenic link between psoriasis and CVDs may also have a genetic basis. Mendelian randomization studies suggest a shared genetic predisposition for psoriasis and CVDs, indicating that the relationship is not solely due to lifestyle or traditional risk factors. This genetic overlap underscores the importance of considering psoriasis as a systemic disease with multi-organ involvement [133, 134].

Furthermore, the vascular endothelium plays a crucial role in linking inflammation and coagulation, being vital in regulating both processes [39]. In normal conditions, the endothelium maintains anti-inflammatory and anti-thrombotic properties. On the contrary, in case of pro-inflammatory signals, endothelial damage results in endothelial cell activation. Therefore, endothelium increases the expression of cell adhesion molecules (CAMs) with inflammatory mediators, and procoagulant factors. This breakdown in endothelial function can lead to a prothrombotic, proinflammatory state also with endothelial dysfunction resulting in increased oxidative stress and decreased nitric oxide bioavailability [39].

Cardiovascular risk stratification in patients with psoriasis is challenging. Traditional risk assessment tools like the Framingham risk score may underestimate the actual risk in these patients because they do not account for chronic inflammation. Some guidelines recommend applying a 1.5 multiplier to traditional risk scores for patients with psoriasis or utilizing advanced imaging techniques, such as carotid ultrasound or coronary artery calcium scoring, to detect subclinical atherosclerosis [136, 137].

Therapeutic strategies for managing cardiovascular risk in psoriasis involve both lifestyle modifications and pharmacologic interventions. Statins, for example, are beneficial not only for lipid lowering but also for their anti-inflammatory effects, potentially improving psoriasis severity [136, 137]. Biologic therapies targeting TNF-α, IL- 17, and IL- 23 have shown promise in reducing systemic inflammation and may indirectly lower cardiovascular risk. However, certain treatments, such as cyclosporine and acitretin, may exacerbate hypertension or dyslipidemia and require careful monitoring [137].

In conclusion, psoriasis is a systemic inflammatory disease that significantly increases the risk of cardiovascular morbidity and mortality [133, 137]. The interplay between chronic inflammation, metabolic abnormalities, and genetic factors contributes to this heightened risk [39, 135]. Effective management requires an integrated approach that addresses both dermatologic symptoms and cardiovascular health, emphasizing the importance of multidisciplinary care [137]. Future research should focus on elucidating the mechanisms linking psoriasis and CVDs and optimizing risk stratification and prevention strategies for affected individuals.

#### Take Home Message

Psoriasis is not just a skin disease but a systemic inflammatory condition that significantly increases cardiovascular risk. Chronic inflammation, metabolic disturbances, and genetic predisposition contribute to endothelial dysfunction, atherosclerosis, and cardiovascular events. Traditional risk scores may underestimate this risk, highlighting the need for enhanced screening and a multidisciplinary approach. Effective management should combine lifestyle interventions, statins, and biologic therapies while carefully monitoring treatments that may worsen cardiovascular risk.

### The Role of Nutrition in Psoriasis

Psoriasis is a multifactorial disease influenced by genetic, immune, and environmental factors, with lifestyle and diet playing a significant role in disease severity and outcomes [138]. Individuals with psoriasis often exhibit higher total energy intake, increased consumption of saturated fats and simple carbohydrates, and lower fiber intake compared to controls. Notably, a higher fiber intake is associated with reduced psoriasis severity [138].

Metabolic disorders and obesity contribute to both the onset and exacerbation of psoriasis. Dietary modifications, including caloric restriction and weight loss through tailored hypocaloric diets or bariatric surgery, have been shown to significantly improve Psoriasis Area Severity Index (PASI) scores and enhance therapeutic responses [139]. Additionally, lifestyle changes such as physical activity and dietary education help sustain these benefits [140].

Several dietary patterns have shown benefits for psoriasis severity. Given the shared genetic susceptibility between celiac disease and psoriasis, gluten elimination has been shown to improve psoriasis lesions in individuals with celiac disease or gluten-specific antibodies, though no significant effects were observed in those without these antibodies [141, 142]. The vegetarian diet, characterized by a high intake of vegetables, fruits, legumes, nuts, and cereals while limiting meat, provides anti-inflammatory and antioxidant benefits due to its low saturated fat and arachidonic acid content and high fiber, vitamin, and omega- 3 fatty acid levels [143]. Similar considerations may apply to vegan, plant-based, such as the MedDiet, and DASH diets [57, 143].

Specific nutrients have been investigated for their potential anti-inflammatory and antioxidant effects in psoriasis [144]. While fish oil, zinc, selenium, curcumin, tryptophan, folate, cobalamin, and vitamin E have been studied, there is insufficient evidence to recommend specific supplementation [144]. Vitamin D plays an immunomodulatory role and is involved in epidermal differentiation. Its deficiency is common in individuals with obesity and psoriasis, and while supplementation may be recommended in cases of deficiency, its role in prevention and treatment remains unvalidated [145]. Omega- 3 polyunsaturated fatty acids (PUFAs), particularly eicosapentaenoic acid (EPA) and docosahexaenoic acid (DHA), have shown potential benefits in improving psoriasis symptoms, though studies vary in dosage and duration [146].

Alcohol consumption is a recognized trigger for psoriasis and is associated with increased disease severity [147]. A meta-analysis indicated that each additional gram of daily alcohol intake increases psoriasis risk by 4% [147]. Emerging evidence suggests that gut microbiota dysbiosis, characterized by an increased *Firmicutes/Bacteroides* ratio and reduced microbial diversity, may contribute to psoriasis pathogenesis by promoting systemic inflammation. Probiotic and prebiotic interventions, or symbiotic, are promising areas for future research [148].

Complementary and alternative therapies, including botanical extracts and oral supplements, have been explored for their anti-inflammatory and antioxidant properties [149]. However, results remain inconsistent, and further studies are needed to assess the efficacy of polyphenols, coenzyme Q10, selenium, and zinc in psoriasis management [149].

In conclusion, a healthy lifestyle incorporating a personalized anti-inflammatory diet and regular physical activity is recommended to improve psoriasis severity, enhance response to therapy, and support quality of life. These non-pharmacologic strategies should be considered adjuncts to standard psoriasis management.

#### Take Home Message

A personalized anti-inflammatory diet and regular physical activity play a crucial role in managing psoriasis severity and improving treatment response. Weight loss, Mediterranean and vegetarian diets, and specific nutrients like omega- 3 PUFAs may offer benefits, while alcohol consumption and gut microbiota imbalances can exacerbate symptoms. Non-pharmacologic strategies should be integrated into standard disease management to enhance overall outcomes.

### Impact of the Mediterranean Diet on Psoriasis

Observational studies have shown that individuals with psoriasis are reportedly less adherent to the MedDiet [150–153]. In addition, significant inverse associations between psoriasis severity indexes and adherence to the MedDiet have been reported [152–154]. However, some studies have not found a significant association between adherence to the MedDiet and psoriasis severity [150] (Table 3). Of note, two studies have examined the role of individual components of the MedDiet on psoriasis severity [151, 152]. Barrea et al. conducted a case-controlled, cross-sectional study in 62 individuals with mild-to-severe psoriasis (BMI 31.6 ± 5.1 kg/m^2^), treatment naïve, identifying olive oil and fish consumption as individual predictors of PASI [151]. In addition, fruit and vegetable intake were negatively correlated with PASI [151]. Similarly, Korovesi et al. reported that legumes and extra virgin olive oil were inversely associated with PASI, while dairy consumption was positively associated with severity in 69 individuals with mild-to-severe psoriasis (BMI 28.9 ± 0.7 kg/m^2^), treatment naïve [152]. These findings suggest that specific MedDiet components may exert differential effects on psoriasis severity.

**Table 3:** Clinical studies on the Mediterranean Diet and the Very Low-Energy Ketogenic Therapy in individuals with psoriasis

Authors	Year	Study design	Study population	Type of diet	Duration	Severity index assessed	Results	Main conclusions
Aryanian et al [150]	2024	Case–controlled, cross-sectional study	71 cases with mild-to-severe psoriasis (aged 38.2 ± 12.6 years); 71 age and sex matched healthy controls	MedDiet (PREDIMED 14-item questionnaire was used to assess adherence to the MedDiet)	n/a	PASI	Adherence to the MedDiet of individuals with psoriasis was significantly lower than controls (*p* = 0.004). The consumption of fruit and fish in individuals with psoriasis was significantly lower and consumption of red meat was significantly higher compared to controls. No significant relationship was found between the severity of the disease and adherence to the MedDiet	A significant difference between individuals with psoriasis and controls following the MedDiet might be indicative of the relationship between diet and psoriasis and the potential benefits of this type of diet due to its anti‐inflammatory properties
Molina-Leyva et al [154]	2019	Cross-sectional study	92 individuals with plaque psoriasis in systemic treatment	MedDiet (PREDIMED 14-item questionnaire was used to assess adherence to the MedDiet)	n/a	VAS, PASI, BSA, PGA	The severity of psoriasis, both PASI (*p* = 0.007), BSA (*p* = 0.009), PGA (*p* = 0.01) and VAS (*p* = 0.004) were lower in individuals with higher adherence to the MedDiet	Higher adherence to the MedDiet is associated with a lower severity of psoriasis, both objective and subjective
Korovesi et al [152]	2019	Case–controlled, cross-sectional study	69 cases with moderate-to-severe psoriasis (aged 47.7 ± 13.3 years; BMI 28.9 ± 0.7 kg/m^2^), treatment naive; 69 age, sex and BMI matched healthy controls	MedDiet (MedDietScore 12-items questionnaire was used to assess adherence to the MedDiet)	n/a	PASI, DLQI	Individuals with psoriasis showed lower adherence to the MedDiet (*p* < 0.001) compared to controls. Adherence to the MedDiet was inversely associated with psoriasis risk above and beyond age, sex, and BMI (OR: 0.34, 95% CI: 0.13–0.92, *p* = 0.03). MedDietScore correlated negatively with PASI (r = − 0.39, *p* = 0.001) and DLQI (r = − 0.41, *p* < 0.001), and CRP (r = − 0.37, *p* = 0.001). Among the items of the MedDietScore, PASI was inversely associated with legumes, fish, and EVOO consumption (*p* < 0.05) and positively associated with dairy products (*p* = 0.002). MedDietScore was a significant negative predictor of PASI (*p* = 0.02) and DLQI (*p* = 0.06, of borderline significance) adjusting for age, sex, BMI, and CRP	Despite the modest sample size, the results of the study suggested an independent inverse association between the adherence to the MedDiet and psoriasis occurrence, severity, and quality of life
Phan et al [153]	2018	Cross-sectional study (based on a web-based questionnaire)	35.735 individuals with psoriasis classified in 3 groups: severe psoriasis, non-severe psoriasis, and no psoriasis (based on hospitalization, systemic treatment, or self-reported severity) aged 47.5 ± 14.0 years	MedDiet (MEDI-LITE 9-items questionnaire was used to assess adherence to the MedDiet)	n/a	None	After adjustment for confounding factors, a significant inverse relationship was found between the MEDI-LITE score and having severe psoriasis: OR, 0.71; 95% CI, 0.55–0.92 for the MEDI-LITE score’s second tertile (score of 8 to 9); and OR, 0.78; 95% CI, 0.59–1.01 for the third tertile (score of 10 to 18)	Individuals with severe psoriasis displayed low levels of adherence to the MedDiet; this finding supports the hypothesis that the MedDiet may slow the progression of psoriasis
Barrea et al [151]	2015	Case–controlled, cross-sectional study	62 cases with mild-to-severe psoriasis (aged 50.2 ± 10.5 years; BMI 31.6 ± 5.1 kg/m^2^), treatment naïve; 62 age, sex and BMI matched healthy controls	MedDiet (PREDIMED 14-item questionnaire was used to assess adherence to the MedDiet)	n/a	PASI	PREDIMED score was lower in individuals with psoriasis compared to controls (*p* = 0.003). PASI score and CRP were significantly associated with the dietary components included in the PREDIMED questionnaire or with the PREDIMED score. At multiple regression analysis, the major predictor of PASI score were fat mass and PREDIMED score (R^2^ = 0.599, β = − 0.296, *p* = 0.007) Among all items of the PREDIMED questionnaire, EVOO (R^2^ = 0.548, β = − 0.741, *p* < 0.001), and fish consumption (R^2^ = 0.139, β = − 0.372, *p* = 0.005) have an independent predictive value for PASI score	This was the first study to evaluate the association between adherence to the MedDiet and the severity of psoriasis
Castaldo et al [158]	2020	Single-arm, open-label trial	37 drug-naïve adults (43.1 ± 13.8 years) with stable chronic plaque psoriasis, with overweight or obesity	Protein-sparing, VLEKT (< 500 kcal/day, 10–20 g of carbohydrate/day, 20—30 g of lipids/day, 1.2 g protein/kg of IBW)	4 weeks	BSA involved and PASI	Significant reduction of BSA involved (− 10.1%, *p* < 0.001) and PASI score (− 7.2, *p* < 0.001)	The effects of VLEKT on improving clinical outcomes of psoriasis were shown to be independent of disease severity and overweight at baseline. These effects are attributed to the reduction in VAT (mean reduction of 4 mm in AMFT, *p* < 0.001), which in turn reduces the pro-inflammatory stimulus, as shown by the observation of reduced TNF-α, INF-γ, IL- 1β and IL- 2 levels (*p* < 0.001 for all)
Castaldo et al [157]	2021	Intervention	30 patients with plaque psoriasis, overweight, aged 18—65 years old	Very-low-calorie, protein-based diet (< 500 kcal/day, 10—20 g of carbohydrate/day, 20—30 g of lipids/day, 1.4 g protein/kg of IBW)	4 weeks	PASI	Improvements in all parameters related to psoriasis (PASI reduction of about 50%)	4 weeks of a VLEKT is able to reduce the progression of the disease by significantly reducing disease-related clinical scores such as PASI, VAS pain and VAS pruritus. This is associated with an improvement in clinical and biochemical parameters related to psoriasis, such as levels of folic acid, vitamin B12, calcium, bilirubin, cortisol, LDL, total cholesterol and pro-inflammatory cytokines. These effects would primarily be due to an action of the VLEKT on the reduction of VAT

*MedDiet* Mediterranean diet; *PREDIMED* PREvención con DIeta MEDiterránea; *PASI* Psoriasis Area and Severity Index; *VAS* Visual Analog Scale; *BSA* Body Surface Area; *PGA* Practitioner Global Assessment; *DLQI* Dermatology Life Quality Index; *OR* Odds ratio; *CI* confidence interval; *CRP* C-reactive protein; *EVOO* Extra virgin olive oil; *MEDI-LITE* Mediterranean diet based on the literature; *VLEKT* Very-Low Energy Ketogenic Therapy; *IBW* Ideal Body Weight; *VAT* Visceral Adipose Tissue; *AMFT* Aorto-Mesenteric Fat Thickness; *TNF-α* Tumor Necrosis Factor-α; *IFN-γ* Interferon-γ; *IL* Interleukin; *LDL* low-density lipoprotein cholesterol

The beneficial effects of the MedDiet on psoriasis may be attributed to its anti-inflammatory and antioxidant properties [155]. The diet is rich in polyphenols, omega- 3 fatty acids, and fiber, which can modulate systemic inflammation by reducing pro-inflammatory cytokines such as TNF-α, IL- 6, and IL- 17—key mediators in psoriasis pathogenesis. Extra virgin olive oil and nuts provide monounsaturated and polyunsaturated fats, which have been shown to improve endothelial function and reduce oxidative stress, factors implicated in chronic inflammatory diseases like psoriasis. Additionally, high fiber intake from legumes, fruits, and vegetables promotes gut microbiota diversity, leading to the production of short-chain fatty acids with immunomodulatory effects. These mechanisms collectively suggest that adherence to the MedDiet may help in mitigating psoriasis severity and improving overall skin health [155].

Further research is needed to clarify the protective effects of the MedDiet on psoriasis. Its heterogeneity makes it challenging to identify specific beneficial components. Standardized adherence assessments and large-scale RCTs are essential to establish causality and better define its impact on psoriasis severity.

#### Take Home Message

The MedDiet’s anti-inflammatory and antioxidant properties, driven by polyphenols, omega- 3, and fiber, may help reduce psoriasis severity by modulating key inflammatory pathways. Specific components like extra virgin olive oil, fish, legumes, and high fiber intake have shown beneficial associations. Further research is needed to confirm these effects through standardized assessments and large-scale RCTs.

### The Impact of the Ketogenic Diet on Psoriasis

Despite the paucity of evidence in this regard, the rationale behind the use of VLEKT in the management of psoriasis is well established and lies essentially in the antioxidant and anti-inflammatory potential of this MNT [156]. However, compared to acne and HS, for which only one study on the effects of VLEKT is available, two Italian intervention studies conducted by the same research group investigated the role of ketogenic therapy in adult, systemic drug naïve patients with stable chronic plaque psoriasis, and presence of obesity or overweight, albeit in a limited number of subjects (30 and 37) [157, 158] (Table 3). In both studies, ketogenic therapy had the nutritional characteristics of VLEKT [12], although the nutritional plans were not developed according to the KeNuT-SIE protocol [69]. Overall, the studies reported concordant results, demonstrating a significant effect of VLEKT on improving clinical outcomes and psoriasis severity [157, 158]. In particular, in addition to the reduction in PASI, visual analogue scale (VAS) pain and VAS pruritus, a reduction in body surface area involved was also reported in one of the two studies. The main mechanism underlying the observed results lies in the anti-inflammatory effect of VLEKT. In both studies, in fact, significant reductions in TNF-α, INF-γ, IL- 1β and IL- 2 were reported due, plausibly, to a VLEKT-induced reduction in visceral adipose tissue, as demonstrated by the significant reduction in aorto-mesenteric fat thickness. An equally interesting finding observed is the effect of VLEKT on psoriasis-related metabolite levels. Following the period of ketogenic therapy, an improvement in lipid levels (particularly total and LDL-cholesterol), cortisol, bilirubin and microelements (folic acid, vitamin B12, and calcium) was observed. Overall, ketogenic therapy resulted in a significant improvement in the patients'quality of life (as assessed by the DLQI), suggesting this approach as effectively valid in the multifactorial management of the patient with psoriasis [157, 158].

#### Take Home Message

In patients with psoriasis and co-existing overweight/obesity, VLEKT may be a valid strategy for managing the pathological condition. The nutritional characteristics of ketogenic therapy should be as follows: < 500 kcal/day, 10–15 g/day carbohydrates, 20–30 g/day lipids, 1.2–1.4 g/kg ideal body weight *per* day. In order to increase patients'dietary compliance and avoid a negative impact on the gut microbiota, it is suggested to elaborate VLEKT according to the KeNuT-SIE protocol, with the use of replacement meals.

**Fig. 3:**
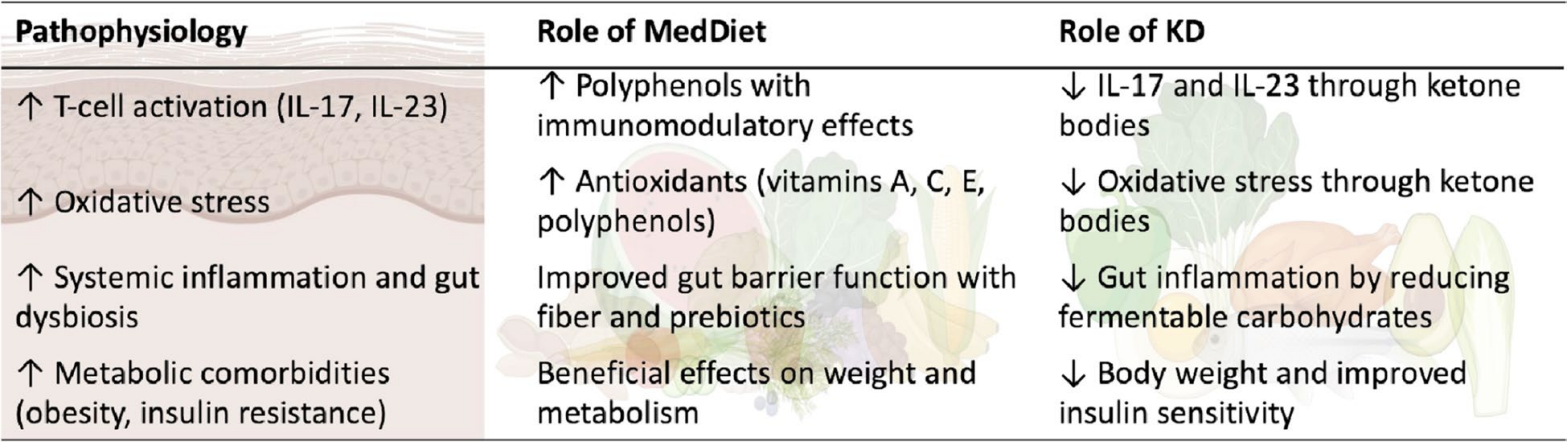
Pathophysiology and role of the MedDiet and ketogenic diet in psoriasis disease, summarizes the pathophysiology and role of the Mediterranean and ketogenic diets in psoriasis disease, *IL, Interleukin; MedDiet, Mediterranean Diet; KD, ketogenic diet*

Figure 3 shows a summary of the pathophysiology and role of the MedDiet and ketogenic diet in psoriasis disease.

## Conclusions

**Fig. 4:**
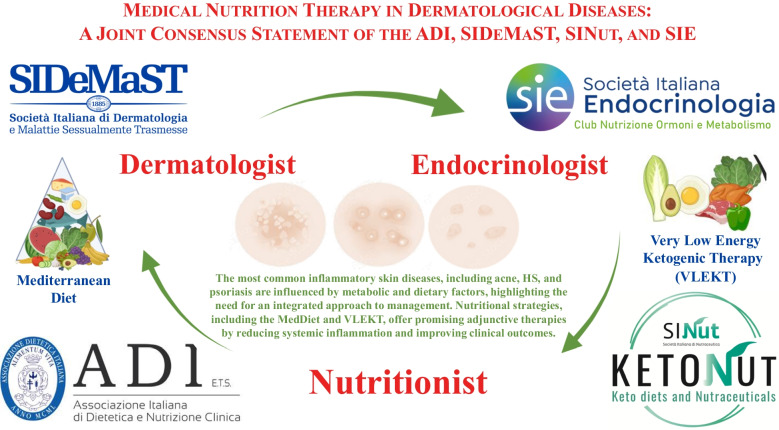
Consensus statement of the ADI, SIDeMaST, SINut, and SIE. The most common inflammatory skin diseases, including acne, HS, and psoriasis are influenced by metabolic and dietary factors, highlighting the need for an integrated approach to management. Nutritional strategies, including the MedDiet and VLEKT, offers promising adjunctive therapies by reducing systemic inflammation and improving clinical outcomes. *ADI, Italian Association of Dietetics and Clinical Nutrition; SIDeMaST, Italian Society of Dermatology and Sexually Transmitted Diseases; SIE, Italian Society of Endocrinology; SINut, Italian Society of Nutraceuticals; VLEKT, Very Low-Energy Ketogenic Therapy*

In this joint *Consensus Statement* of the ADI, SIDeMaST, SINut, and SIE, we report an evidence-based framework for MNT, including the MedDiet and the VLEKT, for the most common inflammatory skin diseases, such as acne, HS, and psoriasis (Fig. 4).

Acne, HS and psoriasis are influenced by obesity, metabolic and dietary factors, highlighting the need for an integrated and multidisciplinary approach in clinical management that includes the dermatologist, the endocrinologist and the nutritionist.

Medical nutrition therapy offers promising results not only in improving obesity, metabolic alterations and cardiovascular risk but, in leading to the reduction of inflammation and oxidative stress, directly and indirectly reduce the clinical severity of the main inflammatory skin diseases. In patients with inflammatory skin diseases, both the MedDiet and the VLEKT should be prescribed only after a proper clinical evaluation by the multidisciplinary team.

While observational studies suggest beneficial associations between dietary patterns and skin diseases severity, the lack of large-scale randomized controlled trials limits definitive conclusions. Future research should prioritize well-designed RCTs to establish causality, refine dietary recommendations, and standardize assessment methods for dietary adherence. Personalized nutrition strategies should be incorporated into routine dermatological care to enhance treatment efficacy and to improve patient quality of life.

## Data Availability

No datasets were generated or analysed during the current study.

## Abbreviations

ADI: Italian Association of Dietetics and Clinical Nutrition
BMI: Body Mass Index
CVD: Cardiovascular Disease
DASH: Dietary Approaches to Stop Hypertension
EPA: Eicosapentaenoic Acid
GLP- 1: Glucagon-Like Peptide-1
HDL: High-Density Lipoprotein
HS: Hidradenitis Suppurativa
IGF- 1: Insulin-Like Growth Factor 1
IL: Interleukin
KeNuT: Ketogenic Nutritional Therapy
LDL: Low-Density Lipoprotein
MedDiet: Mediterranean Diet
MetS: Metabolic Syndrome
MNT: Medical Nutrition Therapy
mTORC1: Mechanistic Target of Rapamycin Complex 1
PASI: Psoriasis Area Severity Index
PUFA: Polyunsaturated Fatty Acid
RCT: Randomized Controlled Trial
ROS: Reactive Oxygen Species
SIE: Italian Society of Endocrinology
SINut: Italian Society of Nutraceuticals
SIDeMaST: Italian Society of Dermatology and Sexually Transmitted Diseases
TNF-α: Tumor Necrosis Factor Alpha
VLEKT: Very Low-Energy Ketogenic Therapy
VLCKD: Very Low-Calorie Ketogenic Diet
